# A prospective study of *Escherichia coli* bloodstream infection among adolescents and adults in northern Tanzania

**DOI:** 10.1093/trstmh/trz111

**Published:** 2019-12-10

**Authors:** Deng B Madut, Matthew P Rubach, Nathaniel Kalengo, Manuela Carugati, Michael J Maze, Anne B Morrissey, Blandina T Mmbaga, Bingileki F Lwezaula, Kajiru G Kilonzo, Venance P Maro, John A Crump

**Affiliations:** 1 Division of Infectious Diseases and International Health, Duke University Medical Center, 2301 Erwin Rd, Durham, NC, 27710, USA; 2 Duke Global Health Institute, Duke University, 310 Trent Dr, Durham, NC, 27710, USA; 3 Kilimanjaro Christian Medical Centre, PO Box 3010, Moshi, Tanzania; 4 Department of Medicine, University of Otago, PO Box 56, Dunedin, 9054, New Zealand; 5 Centre for International Health, University of Otago, PO Box 56, Dunedin, 9054, New Zealand; 6 Kilimanjaro Christian Medical University College, Tumaini University, PO Box 3010, Moshi, Tanzania; 7 Mawenzi Regional Referral Hospital, PO Box 3054, Moshi, Tanzania

**Keywords:** Africa, bacteremia, *Escherichia coli*, risk factors, Tanzania

## Abstract

**Background:**

Characterization of the epidemiology of *Escherichia coli* bloodstream infection (BSI) in sub-Saharan Africa is lacking. We studied patients with *E. coli* BSI in northern Tanzania to describe host risk factors for infection and to describe the antimicrobial susceptibility of isolates.

**Methods:**

Within 24 h of admission, patients presenting with a fever at two hospitals in Moshi, Tanzania, were screened and enrolled. Cases were patients with at least one blood culture yielding *E. coli* and controls were those without *E. coli* isolated from any blood culture. Logistic regression was used to identify host risk factors for *E. coli* BSI.

**Results:**

We analyzed data from 33 cases and 1615 controls enrolled from 2007 through 2018. The median (IQR) age of cases was 47 (34–57) y and 24 (72.7%) were female. *E. coli* BSI was associated with (adjusted OR [aOR], 95% CI) increasing years of age (1.03, 1.01 to 1.05), female gender (2.20, 1.01 to 4.80), abdominal tenderness (2.24, 1.06 to 4.72) and urinary tract infection as a discharge diagnosis (3.71, 1.61 to 8.52). Of 31 isolates with antimicrobial susceptibility results, the prevalence of resistance was ampicillin 29 (93.6%), ceftriaxone three (9.7%), ciprofloxacin five (16.1%), gentamicin seven (22.6%) and trimethoprim-sulfamethoxazole 31 (100.0%).

**Conclusions:**

In Tanzania, host risk factors for *E. coli* BSI were similar to those reported in high-resource settings and resistance to key antimicrobials was common.

## Introduction

*Escherichia coli* is a leading cause of bloodstream infections (BSIs) globally and a major public health concern.^1^^,^^2^ This concern is reinforced by the increasing global incidence of *E. coli* BSI from strains resistant to multiple antimicrobial classes.^3^ The public health threat posed by invasive *E. coli* infections warrants continued epidemiologic surveillance and justifies current efforts to develop vaccines and other prevention interventions.^4^ Similar to other regions of the world, *E. coli* has been identified as a leading cause of BSI in the countries of sub-Saharan Africa (sSA).^5^ Despite this, there are limited data describing the epidemiology of *E. coli* BSI in sSA.

Much of our understanding of the epidemiology of *E. coli* BSI comes from studies conducted in high-resource settings. In these settings, multiple reports indicate that *E. coli* BSI frequently occurs as a complication of focal infection of the urinary tract. Older age, manipulation of the urinary tract and comorbid illnesses such as heart disease, liver disease and malignancies are important host risk factors for BSI.^6–9^ The case fatality ratio (CFR) of patients with *E. coli* BSI varies but may be as high as 60%.^10–12^ The heterogeneity in reported CFRs likely reflects differences in baseline characteristics of patient cohorts and the influence of antimicrobial-resistant organisms.

Advances in molecular typing methods have provided additional insights into the epidemiology of *E. coli* BSI.^13^ This work suggests that invasive infections, including those causing BSI, are caused by lineages, or clonal groups, of extraintestinal pathogenic *E. coli* (ExPEC) that possess virulence traits distinct from commensal and diarrhegenic *E. coli*.^14^ While a high degree of genomic diversity exists among ExPEC, studies suggest that a limited number of lineages are responsible for a large majority of all BSIs.^15^^,^^16^ Among these highly successful lineages, a few are of major public health concern given their multidrug-resistant (MDR) phenotype and evidence suggesting possible reservoirs within food animals and environmental sources.^17^ In this respect, sSA countries may provide valuable insights into the epidemiology of invasive ExPEC given that a considerable proportion of the population lives within close proximity to food animals, basic sanitation services are limited in some settings, and human and non-human antimicrobial use remains largely unregulated.^18^

Compared with other regions, the epidemiology of *E. coli* BSI in sSA countries is poorly described. In order to provide insight into the epidemiology, we studied adolescent and adult patients with *E. coli* BSI enrolled in hospital-based fever surveillance studies in Moshi, Tanzania, to describe major host risk factors for infection and antimicrobial susceptibility patterns of isolates.

## Methods

### Study setting

We conducted prospective hospital-based fever surveillance studies at Kilimanjaro Christian Medical Centre (KCMC) and Mawenzi Regional Referral Hospital (MRRH) in Moshi, Tanzania, during the periods of 17 September 2007 through 31 August 2008, 26 September 2011 through 31 May 2014 and 7 September 2016 through 16 September 2018. Results from these studies have been previously described.^19^–^23^ KCMC is a 630-bed zonal referral hospital that serves the regions of Kilimanjaro, Tanga, Arusha, Manyara, Dodoma and Singida in the northern zone of Tanzania. MRRH is a 300-bed regional hospital serving the Kilimanjaro region. Moshi (population >180 000) is the administrative center of the Kilimanjaro region (population >1.6 million) and is situated at an elevation of approximately 890 m above sea level. The climate is tropical, with rainy seasons from March through May and October through December. Malaria transmission is low in the Kilimanjaro region and community HIV prevalence among adults aged 15–49 y is 3.8%.^25^

### Study procedures

Adult and adolescent patients (aged ≥13 y) were enrolled from Monday through Friday in the inpatient units of KCMC and MRRH. All patients admitted to these units were screened and enrolled within 24 h after admission. Patients presenting with a fever were eligible for enrolment. Fever was defined for the period 2007–2008 as inpatients with an oral temperature of ≥38.0°C, and for the periods 2012–2014 and 2016–2018 as inpatients with an oral or tympanic temperature of ≥38.0°C or a history of fever within the previous 72 h.

After obtaining informed consent, a trained study team member conducted a standardized clinical history and physical examination. Data collected included age, gender, temperature, symptoms of dysuria, presence of abdominal tenderness upon physical examination, prior antimicrobial use and healthcare contact prior to the current admission. Dysuria was self-reported and was only collected in the periods of 2007–2008 and 2016–2018. Prior antibacterial and antimalarial use were defined as self-reported usage of these medications for the present illness prior to admission. Questions regarding healthcare contact prior to the current admission differed for each study period. During the period 2007–2008, participants were asked ‘Were you referred from the inpatient department of this or another hospital?’ During the period 2012–2014, participants were asked if they ‘Sought care for this illness at another location prior to presentation at KCMC/MRRH?’ If the participant answered yes, they were asked which healthcare facilities they did visit prior to their current admission. During the period of 2014–2016, participants were asked to list all of the healthcare facilities visited for the present illness prior to the current admission. In addition, they were asked if they were admitted to any of these facilities.

Blood was collected for blood culture, HIV testing and thin and thick smears for blood parasites. Blood cultures were collected within 24 h of admission. Blood culture bottles for the period 2007–2008 were BacT/ALERT Standard Aerobic and Mycobacterial MB (bioMérieux Inc., Durham, NC, USA); for 2012–2014 they were BacT/ALERT Standard Aerobic; and for 2016–2018 they were BacT/ALERT FA Plus with BacT/ALERT Standard Aerobic as back-up in the case of FA Plus running out of stock. Blood cultures were assessed for volume adequacy by measuring the weight before and after inoculation. An adequate blood volume for BacT/Alert Standard Aerobic and FA Plus Aerobic bottles was 8–12 mL and an adequate blood volume for BacT/Alert MB bottles was 4–6 mL. At the time of hospital discharge, a study team member completed a discharge form which included the participant's admission duration in days, discharge diagnoses using International Classification of Diseases coding assigned by non-study clinicians and vital outcome.

### Laboratory methods

Aerobic and mycobacterial bottles were loaded into the BacT/ALERT 3D microbial detection system and incubated for 5 and 42 d, respectively. *E. coli* biochemical identification was performed using API 20E (bioMérieux Inc., Marcy-I'Étoile, France) test strips. Antimicrobial susceptibility testing was performed according to the methods of the Clinical Laboratory Standards Institute (CLSI, Wayne, PA, USA). Susceptibility interpretations were based on the 2019 CLSI guidelines and interpretive criteria, classified as susceptible, intermediate or resistant.^25^

HIV-1 antibody testing methods during the study period of 2007–2008 have been previously described.^19^ During the study period of 2012–2014, HIV infection status was self-reported. During the period of 2016–2018, HIV antibody testing was accompanied by pre- and post-test counseling and was performed on whole blood using the Standard Diagnostics Bioline HIV-1/HIV-2 3.0 test (Standard Diagnostics Inc., Kyonggi-do, South Korea) for screening followed by the Unigold Rapid HIV test (Trinity Biotech, Bray, Ireland) for confirmation.

Malaria infection was assessed by thick and thin blood films stained with Giemsa. Blood films were examined for blood parasites by oil immersion microscopy. Parasite density was determined by standard methods.^26^

### Definitions

Cases were defined as participants with at least one blood culture yielding *E. coli*. Controls were defined as participants without *E. coli* isolated from any blood culture. Cases were classified as hospital-onset *E. coli* BSI if they were referred from or admitted to another hospital prior to their current admission. Thus, no participants in this study were eligible to meet the traditional hospital-onset infection definition of BSI identified 48 h or later after admission.^2^ Cases were classified as community-onset *E. coli* BSI if they reported no hospital contact or admissions prior to their current admission. Cases were classified as indeterminate onset if they reported prior hospital contacts before admission but were not admitted at another hospital or if the available data were insufficient to classify them as community-onset or hospital-onset.

### Statistical analysis

Data analysis was performed using STATA version 15.1 (StataCorp, College Station, TX, USA). Continuous variables are expressed using median and interquartile range (IQR). Categorical variables are expressed as frequencies. Logistic regressions were performed to examine associations of different host risk characteristics with *E. coli* BSI. Variables associated with *E. coli* BSI at p<0.2 in bivariable logistic regressions were selected as possible predictors of *E. coli* BSI in multivariable logistic regressions. A backwards elimination strategy was performed with variables with p<0.05 kept in the final model. All p-values are two-sided and a p<0.05 was considered statistically significant. The population attributable fraction (PAF) and associated 95% confidence interval for modifiable risk factors were further computed based on the adjusted regression analyses. The PAF is the theoretical proportion of *E. coli* BSI cases that would be prevented if a specific host factor was eliminated.^27^

## Results

### Participant characteristics

Of 1648 participants aged ≥13 y who had at least one blood culture collected, 33 (2.0%) had *E. coli* BSI and the remaining 1615 (98.0%) did not have *E. coli* BSI and were assigned as controls. Of participants, 24 (72.7%) cases and 875 (54.2%) controls were female (p=0.040). The median (IQR) age of cases was 47 (34–57) y and of controls was 38 (28–48) y (p=0.008). HIV-infection was identified among eight (29.6%) cases and 462 (35.2%) controls (p=0.548). Among HIV-infected participants, prophylactic use of trimethoprim-sulfamethoxazole was not reported for any cases but was reported among 226 (48.9%) controls. The inpatient mortality among cases was four (12.1%) and that among controls was 121 (7.5%). Among cases, 24 (72.7%) were classified as community-onset, one (3.0%) was classified as hospital-onset and eight (24.2%) had indeterminate onset. Participant characteristics are presented in Table 1.

**Table 1:** Demographic, clinical and laboratory characteristics of hospitalized patients with *Escherichia coli* bloodstream infection compared with patients without *Escherichia coli* bloodstream infection, northern Tanzania, 2007–2018

	Cases (n=33)	Controls (n=1615)	OR	(95% CI)	p
Demographic characteristics					
Age, median y (IQR)	47 (33–57)	38 (28–48)	1.03	(1.01 to 1.05)	0.008^*^
Female gender, n (%)	24 (72.7)	875 (54.2)	2.25	(1.04 to 4.87)	0.040^*^
Admission history and findings					
Duration of illness prior to admission, median days (IQR)	7 (5–14)	7 (3–17)	0.98	(0.97 to 1.01)	NS
Temperature, °C, median (IQR)	38.4 (38–38.9)	38.1 (37.5–38.8)	1.23	(0.86 to 1.74)	NS
Dysuria^a^, n (%)	8 (44.4)	130 (14.7)	4.63	(1.79 to 11.94)	0.002^*^
Abdominal tenderness, n (%)	12 (36.4)	271 (17.0)	2.79	(1.35 to 5.75)	0.005^*^
Prior antibacterial use^b^, n (%)	11 (33.3)	660 (42.0)	0.69	(0.33 to 1.43)	NS
Prior antimalarial use, n (%)	9 (27.3)	519 (32.7)	0.77	(0.35 to 1.67)	NS
Laboratory findings					
Malaria detected by microscopy, n (%)	0 (0)	57 (3.6)			
HIV, n (%)	8 (29.6)	462 (35.2)	0.77	(0.34 to 1.78)	NS
Discharge findings					
Admission duration, median days (IQR)	6 (4–7)	4 (2–7)	1.00	(0.98 to 1.02)	NS
UTI as discharge diagnosis, n (%)	8 (24.2)	113 (7.2)	4.12	(1.82 to 9.34)	0.001^*^
Inpatient deaths, n (%)	4 (12.1)	121 (7.5)	1.69	(0.58 to 4.88)	NS

^*^Statistically significant.

^a^Dysuria symptoms was collected only in period 1 (2007–2008) and period 3 (2016–2018).

^b^Prior antibacterial or antimalarial medications are defined as the use of these medications for the present illness prior to hospital admission. NS: not significant

In this population, *E. coli* was the second most common cause of BSI after *Salmonella enterica* serovar Typhi, which caused 35 (2.1%) BSIs. Of 1968 aerobic blood culture bottles collected during the study periods, fill volume data were available for 1893 (96.2%) and 1680 (88.7%) were classified as adequately filled. Of 321 mycobacterial bottles collected during the study, volume data were available for 314 bottles (97.8%) and 220 (70.1%) were classified as adequately filled.

### Risk factors for E. coli bloodstream infection

Bivariable logistic regression results are presented in Table 1. In multivariable analysis (Table 2), increasing age (adjusted OR [aOR], 1.03, 95% CI, 1.01 to 1.05; p=0.024), female gender (aOR, 2.20, 95% CI, 1.01 to 4.80; p=0.048), abdominal tenderness (aOR, 2.58, 95% CI, 1.06 to 4.72; p=0.011; PAF, 22.3%, 95% CI, 11.2% to 31.9%) and urinary tract infection (UTI) as a discharge diagnosis (aOR, 3.68, 95% CI, 1.61 to 8.52; p=0.002; PAF, 17.7%, 95% CI 12.1% to 23.0%) were associated with increased odds of *E. coli* BSI.

**Table 2:** Multivariable analysis of risk factors for *Escherichia coli* bloodstream infection among hospitalized patients with a febrile illness, northern Tanzania, 2007–2018

	Odds Ratio	(95% CI)	p-value	PAF, %	(95% CI)
Age (y)	1.02	(1.01 to 1.05)	0.024^*^		
Female gender	2.20	(1.01 to 4.80)	0.048^*^		
Abdominal tenderness	2.58	(1.06 to 4.72)	0.011^*^	22.3	(11.2 to 31.9)
UTI as discharge diagnosis	3.68	(1.61 to 8.52)	0.002^*^	17.7	(12.0 to 23.0)

^*^Statistically significant.

PAF: population attributable fraction; UTI: urinary tract infection.

**Table 3:** Antimicrobial susceptibility of 31 *Escherichia coli* isolates from hospitalized patients with bloodstream infection, northern Tanzania, 2007–2018

	Susceptible	Intermediate	Resistant
Ampicillin, n (%)	2 (6.5)	0 (0)	29 (93.6)
Ceftriaxone, n (%)	28 (90.3)	0 (0)	3 (9.7)
Ciprofloxacin, n (%)	25 (80.7)	1 (3.2)	5 (16.1)
Gentamicin, n (%)	24 (77.4)	0 (0)	7 (22.6)
Trimethoprim-sulfamethoxazole, n (%)	0 (0)	0 (0)	31 (100.0)

### Antimicrobial susceptibility

Of 33 isolates, complete antimicrobial susceptibility testing results (Table 3) were available for 31 (93.9%). Among isolates with antimicrobial susceptibility results, 29 (93.6%) were resistant to ampicillin, three (9.7%) ceftriaxone, five (16.1%) ciprofloxacin, seven (22.6%) gentamicin and 31 (100.0%) to trimethoprim-sulfamethoxazole. Of the three isolates resistant to ceftriaxone, two (66.7%) were from patients enrolled during the study period of 2016–2018, and of the five isolates resistant to ciprofloxacin, four (80.0%) were from patients enrolled during the study period of 2016–2018.

## Discussion

We demonstrated that *E. coli* was a common cause of BSI in a cohort of febrile hospitalized patients in Moshi, Tanzania. On multivariable analysis, *E. coli* BSI was associated with increasing age, female gender, abdominal tenderness and UTI as a discharge diagnosis. In addition, we found that a large proportion of isolates were resistant to antibacterials commonly available in the community and hospital setting in Tanzania.

The host risk factors associated with *E. coli* BSI in our Tanzania study were similar to previous reports from high-resource settings. Numerous studies have found an association between *E. coli* BSI and increasing age.^7–9^ Population aging is hypothesized to be one factor that has contributed to observed increases in *E. coli* globally.^28^ Previous research indicates that age may influence the gender differences observed in the risk for *E. coli* BSI. Much of the excess risk in women occurs from ages 1 through 60 y; while among men, those aged >60 y are at the highest risk of *E. coli* BSI.^7^^,^^12^ Irrespective of age and gender, studies shows that the most common anatomic source of *E. coli* BSI is the urinary tract.^9^^,^^29^ The association between a discharge diagnosis of UTI and *E. coli* BSI in the Tanzania population suggests that the urinary tract likely serves as an important anatomic source of BSI. While our results indicate that appropriate treatment of UTIs in the community setting may reduce the number of *E. coli* BSIs, more work is needed to determine other potential sources beyond the urinary tract as UTI explained only a minority of BSIs in our study.

Abdominal tenderness was associated with *E. coli* BSI in our study. Previous studies indicate that following the urinary tract, primary infection of the hepatobiliary or gastrointestinal tract is a leading source of *E. coli* BSI.^7^^,^^11^ Thus, the association between abdominal tenderness and *E. coli* BSI in our study may suggest intra-abdominal infections as an important source of BSI. However, the lack of complementary imaging studies or cultures from potential intra-abdominal foci of infection prevents accurate anatomic attribution, since abdominal tenderness on physical examination could be an indication of underlying intra-abdominal infection or UTI.

HIV infection was common among participants in our study but we found no association between HIV infection and *E. coli* BSI. Previous reports indicate that HIV-infected patients in sSA countries are at an increased risk of bacterial BSI, notably from non-typhoidal serovars of *S. enterica* and mycobacteria.^3^^,^^15^ While *E. coli* has been found to be a common pathogen among HIV-infected patients presenting with BSIs, studies have not consistently shown a higher risk for *E. coli* BSI among HIV-infected compared with individuals uninfected with HIV.^5^^,^^30^ Notably, self-reported use of prophylactic trimethoprim-sulfamethoxazole was higher among HIV-infected controls in our population in comparison with HIV-infected cases. The use of trimethoprim-sulfamethoxazole has been shown to decrease the risk of invasive bacterial diseases, including BSIs.^31^^,^^32^

Consistent with low malaria transmission in our study setting, malaria prevalence was low in our study: none of 30 cases of *E. coli* BSI and only 57 (3.6%) of controls had malaria infection. Among African children, malaria infection has been associated with an increased risk of gram-negative BSI.^33^^,^^34^ However, the evidence for an association between malaria infection and gram-negative BSI among adolescents and adults is not as strong.^35^^,^^36^ The low prevalence of malaria among participants in our study precluded further evaluation of an association between malaria and *E. coli* BSI.

Reports on the antimicrobial resistance (AMR) for *E. coli* BSI isolates from sSA countries are sparse.^5^^,^^37^ We found a high proportion of isolates resistant to ampicillin and trimethoprim-sulfamethoxazole, both commonly available antibacterials in the community setting. If prompt diagnosis and appropriate treatment of UTI is one potential strategy to reduce the burden of *E. coli* BSI, our reported resistance to ampicillin and trimethoprim-sulfamethoxazole suggest that some patients in our setting may receive inappropriate antimicrobial therapy in the community. Similarly, the high proportion of isolates resistant to gentamicin is concerning given that ampicillin and gentamicin are included among empiric treatment regimens suggested for severe febrile illness or sepsis in low-resource settings.^38^ This implies that a substantial proportion of patients with *E. coli* BSI would be expected to fail empiric treatment if this regimen were used in our study population. Overall, these findings highlight the need for continued AMR monitoring to inform and refine local, regional and international clinical management guidelines.

Resistance to ceftriaxone and ciprofloxacin was low during the first two study periods but appeared in the most recent study period. Globally, resistance to cephalosporins and fluoroquinolones among *E. coli* BSI isolates is increasing.^3^^,^^15^ The existing evidence suggest that this increase in the prevalence of resistance is largely driven by one *E. coli* lineage, sequence type (ST) 131.^39^ Within the past decade, this lineage has emerged from relative obscurity to become a major cause of MDR *E. coli* BSI globally. Molecular typing studies from Tanzania have found carriage of *E. coli* ST 131, as well as other MDR *E. coli* strains, among community dwellers and domestic and companion animals.^40^ Whether the appearance of ceftriaxone and ciprofloxacin resistance in the last study period represents the introduction of *E. coli* ST 131 or another resistant clone into our study population is unclear. Molecular typing of our isolates may be an important next step to further understanding the epidemiology of *E. coli* BSI in our population. Such work, in combination with previous molecular typing studies performed in the community setting in Tanzania, has the potential to provide important insights into pathogen-host relationships, reservoirs and transmission pathways of relevant *E. coli* sequence types.

Our study has several limitations. First, although there were a large number of patients enrolled in the study, we identified only 33 participants with *E. coli* BSI. Given the limited number of cases, we recognize that our regression analysis is subject to overfitting.^41^ While caution should be exercised when interpreting multivariable regression models with an event per predictor variable (EPV) of less than 10, models containing 5–9 EPV can be valid, particularly in the setting of highly significant and plausible associations.^42^ Second, all enrolments of febrile adolescents and adults were conducted in the general medicine wards of the two hospitals. Thus, it is possible that we have underestimated the prevalence of intra-abdominal sources of *E. coli* BSI that might have presented to surgical services rather than be admitted to general medicine inpatient units. Third, only one aerobic blood culture bottle was collected for the majority of participants in this study. While the majority of these bottles had optimal blood volumes, the collection of additional blood culture bottles would have increased the sensitivity to detect BSIs. Hence, these data are subject to misclassification of cases as controls. However, such misclassification would bias towards the null and thus does not invalidate the risk factors for *E. coli* BSI that we have described. Fourth, we expected that a high proportion of our participants would have community-onset BSIs because all blood cultures were collected within 24 h of admission. Even among the eight participants for which a setting of BSI acquisition could not be determined, we suspect a large proportion of these participants likely had community-onset BSIs because most healthcare interactions prior to their present admission likely occurred in facilities in which invasive interventions would not be performed. In this respect, the results of our study are best generalizable to community-onset rather than hospital-onset *E. coli* BSI. Finally, HIV status was self-reported during the study period 2012–2014. Because self-reported HIV status may be subject to social-desirability bias, we may have underestimated the prevalence of HIV in this population. However, it is unknown if biased self-reporting of HIV status differed between cases and controls.

In summary, *E. coli* is a major cause of BSI globally and was the second most common cause of BSI in cohorts of febrile patients in northern Tanzania spanning from 2007–2018. We found that *E. coli* BSI was associated with risk factors similar to those observed in high-resource settings. In addition, *E. coli* isolates from our study were frequently resistant to antibacterials commonly prescribed in both the community and hospital setting, highlighting the need for AMR surveillance in sSA countries in order to improve clinical management guidelines. Given the prevalence of *E. coli* BSI in sSA countries and globally, further research is needed to explore other major host factors and to understand reservoirs, sources and modes of transmission for *E. coli* strains causing BSI in sSA.

## Notes

Presented in part: 67^th^ Annual Meeting of the American Society of Tropical Medicine and Hygiene, October 28-November 1, 2018, New Orleans, LA, United States of America (Abstract LB-5401).
